# Editorial: Role of microbes in ocular surface health and diseases

**DOI:** 10.3389/fcimb.2024.1462752

**Published:** 2024-08-28

**Authors:** Poonam Mudgil, Vishal Jhanji

**Affiliations:** ^1^ School of Rural Medicine, Charles Sturt University, Orange, NSW, Australia; ^2^ Department of Ophthalmology, University of Pittsburgh, Pittsburgh, PA, United States

**Keywords:** ocular surface microbiota, ocular surface infections, ocular surface health, innate ocular defense, ocular surface homeostasis, *Pseudomonas aeruginosa*, *Staphylococcus aureus*, keratitis

The ocular surface is a dynamic mucosal milieu that is constantly exposed to external environmental influences. Innate host defense mechanisms including physical barriers, antimicrobial chemicals, and immune cells protect it from infections and diseases. In addition, microflora of the ocular surface influences both its health and disease status. The composition of the ocular surface microflora is influenced by intrinsic and extrinsic factors (Patel et al., 2023). Bacteria primarily belonging to Proteobacteria, Actinobacteria, and Firmicutes are normal residents of the healthy ocular surface (Peter et al., 2023). Characterization of ocular microflora is advancing with the use of molecular methods and genome sequencing (Chowdhary et al., 2023). Resident commensal microflora conserves ocular surface health, maintains homeostasis, and interacts with the host immune system to prevent infections (Lai et al., 2024; Zilliox and Bouchard, 2023). Alterations in commensal microflora are associated with ocular surface diseases including vision-threatening keratitis, dry eye disease, meibomian gland dysfunction, blepharitis, conjunctivitis, trachoma, and contact lens-related infections (Chang and Winn, 2023; Ozkan et al., 2023; Schlegel et al., 2023; Shivaji, 2024). Targeting and manipulating ocular microflora may help in therapeutic interventions for ocular surface diseases (Hernández-Zulueta et al., 2023; Lai et al., 2024).

This Research Topic gathers seven papers in the progressively advancing field of ocular microflora and its role in the homeostasis and pathogenesis of the ocular surface and its innate defense. One article discusses the characterization of ocular surface microorganisms. Two articles focus on *Pseudomonas aeruginosa*-induced keratitis, with one showing effects of particulate matter and the other reporting on sheen positive isolates. Bacterial virulence factors and emerging therapies for keratitis are discussed in one article. Differential expression of genes in corneal infections is shown in one article, and another article reviews cases of sight-threatening post-keratoplasty infectious keratitis. One article describes the innate defense of the ocular surface via conjunctival goblet cells.

Herzog et al. address the challenges in the characterization of ocular surface microbiome. The microbial composition and relative abundance of microorganisms are dependent on the type of DNA extraction method and the profiling tool used but not on the swab sampling method. The study highlights the importance of considering these factors for studying microbiomes of sparsely colonized sites such as the ocular surface for better reliability of data and provides potential sources of bias in analyzing microbial composition of low abundance on the ocular surface.

Somayajulu et al. report the adverse effects of particulate matter with a diameter of <10 μm (PM_10_) in *P. aeruginosa*-induced keratitis and the protective effects of SKQ1, a mitochondria-specific antioxidant. By exploring the mechanism of early perforation and thinning of cornea with *P. aeruginosa* infection after PM_10_ exposure, they report that PM_10_ exposure worsens the defense mechanism by oxidative stress, inflammation, and susceptibility to infection, and SKQ1 protects by reversing the adverse effects of PM_10_ via mitochondria-targeted antioxidant effects.

Shah and Wozniak review the pathogenesis of two common causative organisms, *S. aureus* and *P. aeruginosa*, and novel therapies for keratitis. Virulence factors of *S. aureus* include toxins (α, β, and γ), Panton–Valentine leucocidin, fibronectin binding protein, collagen-adhesion binding protein, StaphopainA, extracellular adherence protein, and Staphylococcal enterotoxins. Virulence factors of *P. aeruginosa* include serine protease, *Pseudomonas* protease IV, exotoxins (*exoS*, *exoU*, and *exoT*), *P. aeruginosa* small protease, elastases, and extracellular factors (lipopolysaccharide). Emerging therapies for keratitis include new antibiotics combinations, synthetic derivatives and mimetics of natural host defense peptides, adjunctive therapy, monoclonal antibodies, and non-pharmaceutical approaches such as corneal collagen cross-linking, bacteriophage therapy, and plasma therapy.

Ong et al. examine the clinical presentations, predisposing factors, and outcomes of the sight-threatening post-keratoplasty infectious keratitis by retrospectively reviewing 7 years of cases in Nottingham, UK. The disease is an important cause of infectious keratitis and graft failure. They find *Staphylococcus* spp. as the most common microorganisms and identify risk factors to be bullous keratopathy, ocular surface diseases, and suture-related complication. They advocate the use of prophylactic topical antibiotics and early suture removal for reducing the risk of the disease, which normally creates considerable therapeutic challenge.

Investigating the role of conjunctival goblet cells in ocular innate defense, Li et al. have determined that the α-toxin of toxigenic *S. aureus* initiates two mechanisms for the activation of the nod-like receptor P3 inflammasome in conjunctival goblet cells to provide defense against bacterial infection. The first mechanism involves pore formation by α-toxin, allowing bacterial interactions with toll-like receptor 2. The second mechanism involves pore formation in the plasma membrane by α-toxin, causing an influx of Ca^2+^, which activates the secretion of interleukin-1β. Both mechanisms are required to activate inflammasome in conjunctival goblet cells for protecting the ocular surface against infection.

The study of Shanks et al. on microbial keratitis observes an increase in sheen positive *P. aeruginosa* keratitis isolates in a tertiary care hospital in the Eastern United States. Most isolates are *lasR* mutants having a defective *LasR* transcription factor that regulates virulence factors. Better visual outcomes in the clinical data indicate that sheen positive isolates are less pathogenic. Their reduced extracellular protease activity also indicates less virulence. The *lasR* mutations are highly variable, but the presence of an identical mutation suggests that endemic mutants cause the disease. They report an increase in *lasR* mutants in the United States, and this may be a general worldwide phenomenon.

Alenezi et al. demonstrate differential expression of genes in corneal infections in their study to understand the pathogenic mechanism of microbial keratitis, particularly the overreactive immune response to infection that causes corneal scarring and vision loss. They report that many genes are upregulated in both the cornea and conjunctiva in infection. Most upregulated genes are linked to immune response, angiogenesis regulation, and apoptotic signaling, suggesting that therapeutic designs to curb certain aspects of the immune response may reduce tissue damage and corneal scarring to prevent blindness in microbial keratitis.

We hope that this Research Topic offers valuable insights into the evolving role of microbes in maintaining health and causing diseases of the ocular surface, as well as the innate antimicrobial defense of the ocular surface. Advances in this field are important not only to better understand and treat ocular surface diseases but also to preserve healthy vision.
